# Signatures of natural selection and ethnic-specific prevalence of *NPC1* pathogenic mutations contributing to obesity and Niemann–Pick disease type C1

**DOI:** 10.1038/s41598-020-75919-4

**Published:** 2020-11-02

**Authors:** Andreea Chiorean, William S. Garver, David Meyre

**Affiliations:** 1 Department of Health Research Methods, Evidence, and Impact, McMaster University, 1280 Main Street West, Michael G. DeGroote Centre for Learning and Discovery Room 3205, Hamilton, ON L8S 4K1 Canada; 2 Department of Chemistry and Chemical Biology, University of New Mexico, Albuquerque, NM USA; 3 Department of Pathology and Molecular Medicine, McMaster University, Hamilton, ON Canada

**Keywords:** Evolution, Genetics, Diseases

## Abstract

While homozygous pathogenic mutations in the *NPC1* gene cause Niemann-Pick type C1 disease, heterozygous mutations cause highly-penetrant obesity. We aimed to investigate the prevalence of *NPC1* mutations and their signatures of natural selection in 122,678 exome sequenced participants from six ethnic groups in the Genome Aggregation Database. Pathogenic missense coding mutations were identified by in silico tools and the ClinVar database. Signatures of natural selection were assessed by the probability of *NPC1* being loss-of-function mutation intolerant and Z-scores of observed/expected synonymous and non-synonymous mutation ratios. There was no evidence of negative selection observed for synonymous, non-synonymous and loss-of-function mutations. However, there were significant ethnic differences in the prevalence of heterozygous pathogenic *NPC1* mutations ranging from 0.56% in Ashkenazi Jewish to 3.26% in African/African Americans (5.8-fold difference). Four homozygous carriers of pathogenic *NPC1* mutations were also identified, belonging to the South Asian population. In conclusion, *NPC1* mutations are consistent with a model of balanced selection, where heterozygotes and homozygotes have higher and lower reproductive fitness, respectively. Therefore, *NPC1* heterozygous mutations may account for a substantial and ethnic-dependent percentage of obesity in the general population, while *NPC1* homozygous mutations may be frequent in the South Asian populations and warrants more investigation.

## Introduction

The prevalence of global adult obesity has almost tripled since 1975 to reach 650 million in 2016 according to the World Health Organization. In the past four decades, the number of obese children aged five to 19 has increased tenfold^1^. Obesity is associated with the development of multiple comorbidities^2^. For instance, obesity accounts for 24.3% and 3.6% of the risks for cardiovascular disease and cancer, respectively, the two leading causes of death worldwide^3,4^. Despite several therapeutic modalities (lifestyle and behavioral modifications, medication, bariatric surgery), obesity remains difficult to treat^5^, highlighting the need for new knowledge to predict, prevent, and manage this illness^6,7^. It is well known that obesity results from a complex interplay between biological factors, including genetics, and the environment^6,8^. Twin, family and population studies estimate that 40–75% of body mass index (BMI) variation is explained by genetic determinants^9,10^. Multiple genes responsible for syndromic and non-syndromic monogenic, oligogenic and polygenic forms of obesity have been recently identified in various ethnic groups^6,9,11,12^, including the human Niemann–Pick C1 (*NPC1*) gene^13,14^.

The NPC1 protein mediates the transport of low-density lipoprotein-derived cholesterol and fatty acids from late endosomes/lysosomes into the cytoplasm and other cellular compartments, regulates feedback inhibition of the sterol regulatory binding protein (SREBP) and feedforward activation of the liver X receptor (LXR) pathways^15^. Homozygous or heterozygous compound loss-of-function (LOF) mutations in *NPC1* cause Niemann-Pick type C1 (NPC1) disease, an autosomal recessive lipid storage disorder^16^ with the estimated incidence of 1/92,000^17^. Despite a large spectrum of clinical phenotypes, most patients with NPC1 disease have a life span between 10 and 25 years due to complications resulting from liver failure and neurological degeneration^16^. Similar to humans, a multitude of different NPC1 mouse models have been characterized with NPC1 disease-associated phenotypes including time-dependent cholesterol accumulation in most cells/organs, hepatomegaly, shortened lifespan, and signs of progressive neurologic impairment^18–20^. In 2009, a genome-wide association study (GWAS) identified two common *NPC1* non-synonymous single-nucleotide polymorphisms (SNPs) (rs1805081/H215R and rs1805082/I858V) in linkage disequilibrium that were significantly associated with polygenic morbid obesity in European adult populations but not in European children^13^. More recently, a genome-wide significant association was found between the correlated SNPs rs1805081/H215R, rs1788799/M642I, and rs1805082/I858V) and body mass index (BMI) in predominantly European adult populations^21^. Furthermore, men with rare heterozygous *NPC1* LOF mutations who had children with NPC1 disease had a significantly higher BMI than non-carriers in an East Asian population^14^. Young patients of the same population carrying heterozygous *NPC1* LOF mutations had a fivefold increase in the risk of obesity, although only the associations in men reached significance when the results were stratified by sex^14^. Similar sex-specific associations were found in *NPC1* heterozygous knock-out (*NPC1*^+/*−*^) mice fed a high fat diet (HFD) with significant differences in body weight observed as the mice reached maturity^14^. Other studies also observed a latent weight gain in *NPC1*^+/*−*^ mice fed a HFD^22–25^, supporting the adult onset of obesity observed in human *NPC1* variant carriers^13,21^. More recently, studies have reported that NPC1^+/−^ mice fed a high-fat diet are physiologically characterized with increased liver glycolysis and lipogenesis, and decreased adipose lipolysis through impaired feedback inhibition of the sterol regulatory element binding protein-1 (SREBP-1) pathway. These metabolic disturbances lead to lipid accumulation in the liver and adipose tissue with resultant weight gain in *NPC1*^+/*−*^ mice compared to *NPC1*^+*/*+^ mice fed an identical diet, thereby confirming the gene-diet interaction^23^.

Since NPC1 patients are known to have low reproductive success due to premature death, *NPC1* LOF homozygous/heterozygous compound mutations are prone to negative selection^26,27^. On the contrary and consistent with the ‘thrifty genotype hypothesis’^28^, *NPC1* LOF heterozygous carriers may have been positively selected for during historical periods of famine, considering their increased ability to store fat. This pattern of balancing selection with an heterozygous advantage has been previously described for the widely-accepted ‘malaria hypothesis’^29,30^. Individuals with homozygous mutations for sickle hemoglobin develop sickle cell anemia, a fatal disease if left untreated^29^, while heterozygotes have a protective advantage against the *Plasmodium falciparum* malaria infection^30^.

With respect to rare NPC1 mutations several important gaps of knowledge remain. Specifically, the ethnic specific distribution and signatures of natural selection signature of *NPC1* rare mutations have not been described. Therefore, utilizing the Genome Aggregation Database (gnomAD), we assessed the prevalence and signatures of natural selection of *NPC1* mutations in six ethnic groups (European, Ashkenazi Jewish, East Asian, South Asian, Latino, and African/African American).

## Results

### Signatures of natural selection for *NPC1* mutations

*NPC1* displayed a pLI value of 0 (21 observed versus 62.2 expected number of LOF mutations (Table 1). This signifies that the presence of haploinsufficient LOF mutations (nonsense, splice acceptor, splice donor, and frameshift) in this gene is well-tolerated. No significant enrichment or depletion of missense (637 observed versus 719.4 expected, Z = 1.09) or synonymous (314 observed versus 290.7 expected, Z = -1.07) mutations was observed. Similarly, the observed/expected mutation ratio 90% confidence intervals for LOF, missense and non-synonymous mutations were not indicative of strong mutation intolerance (all 90% confidence interval upper bound values ≥ 0.35, Table 1).

**Table 1:** GnomAD gene constraint for *NPC1.*

Category	Expected No. variants	Observed No. variants	Constraint metrics
Synonymous	290.7	314	Z = − 1.07 o/e = 1.08 (0.98–1.19)
Missense	719.4	637	Z = 1.09 o/e = 0.89 (0.83–0.94)
LOF	62.2	21	pLI = 0.00 o/e = 0.34 (0.43–0.49)

*o/e *observed/expected score, *LOF *loss of function, *pLI *probability of being loss-of-function intolerant.

### Description of the *NPC1* pathogenic mutations identified in gnomAD

We identified 414 distinct rare pathogenic mutations (minor allele frequency < 1% in all ethnic groups) in 1574 heterozygous and four homozygous carriers from six ethnic populations in gnomAD (N = 122,678): 34 frameshift (8.2%), 6 splice acceptor (1.4%), 6 splice donor (1.4%), 13 stop gained (3.1%), and 355 (85.7%) missense mutations. Two of the missense mutations identified as non-pathogenic by SIFT/Polyphen were then identified as pathogenic on ClinVar and included in the analysis. Two-hundred ninety-two missense mutations were classified as non-pathogenic and were not further investigated. Six pathogenic mutations (five missense and one stop gained) were removed from the study because they were only present in the ‘other’ ethnicity group and nine mutations (six missense, two stop lost and two frameshift) with protein consequences not corresponding to isoform 1 were also excluded. More details on these mutations can be found in Supplementary Table 1.

**Figure 1:**
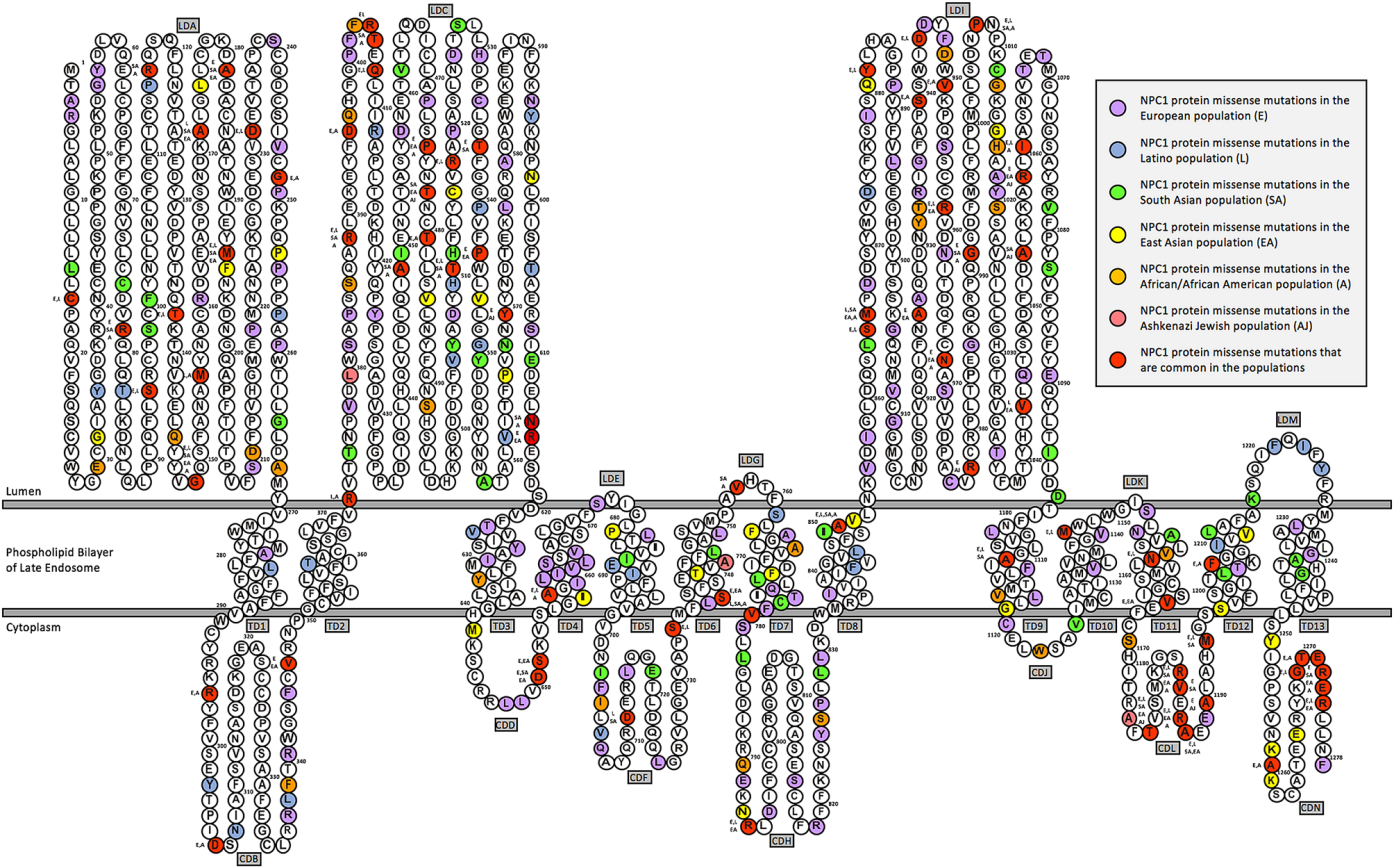
NPC1 protein model of coding pathogenic mutations of six ethnic groups from the gnomAD database. The first amino acid (methionine, Met, or M) positioned at the amino-terminus (left hand side of the figure) is numbered with a superscript on the upper right side of the symbol for methionine (M^1^). The other NPC1 protein amino acids are similarly numbered with a superscript every ten amino acids to facilitate identification of all 1278 amino acids. The location for the 402 coding pathogenic mutations corresponding to ancestral amino acids are color-coded based on the six populations. The red color-coded ancestral amino acids represent locations where a missense mutation for the same or different derived amino acids is present in another population. To indicate these populations, the red color-coded ancestral amino acids are annotated on the middle left side using acronyms for the six populations: Europeans (E), Latino (L), South Asian (SA), East Asian (EA), African/African American (A), and Ashkenazi Jewish (AJ). NPC1 protein domains are designated as luminal domains (LD), cytoplasmic domains (CD), and transmembrane domains (TD) labeled alphanumerically starting from the NPC1 protein amino-terminus and ending at the NPC1 carboxyl-terminus. The sterol-sensing domain (SSD) composed of TD3-TD7.

The location of each pathogenic coding mutation is summarized in an NPC1 protein model (Fig. 1) with the luminal domains (LD), cytoplasmic domains (CD), and transmembrane domains (TD) labeled alphanumerically starting from the NPC1 protein amino-terminus and ending at the NPC1 carboxyl-terminus^31^. Pathogenic missense mutations were categorized by their location in the 27 NPC1 protein domains and are available in Supplementary Table 2. The highest gross number of pathogenic missense mutations was associated with the LDC domain (73 mutations) known to facilitate binding of the NPC2 protein and assorted viruses, the LDI or cysteine-rich domain (69 mutations), LDA (46 mutations) that interacts with NPC2 to accept cholesterol, and the sterol-sensing domain (SSD) composed of TD3-TD7 (38 mutations). Similarly, the highest gross number of individuals possessing mutations were associated with LDC (383 individuals), LDA (304 individuals), LDI (288 individuals), and CDL (128 individuals), but not the SSD composed of TD3-TD7 (74 individuals). The highest percentage of mutant amino acids per domain were represented by CDN (56.67%), CDL (55.17%), TD4 (55.00%), TD7 (55.00%), and CDJ (42.86%) positioned within two of the transmembrane domains of the SSD and the last three cytoplasmic domains for targeting to late-endosomes. To a similar extent, the highest percentage of individuals per domain were represented by CDL (441.38%), CDN (176.67%), CDJ (171.43%), LDC (154.44%), and TD7 (155.00%) positioned within the last three cytoplasmic domains for targeting to late endosomes, the second large luminal domain that binds NPC2 and assorted viruses, and a domain adjacent to the SSD.

### Ethnic differences in the prevalence of *NPC1* pathogenic mutations

Table 2 summarizes the prevalence of heterozygous and homozygous carriers of *NPC1* pathogenic mutations in the six ethnic groups from gnomAD. The coding pathogenic mutations identified in gnomAD are also represented in an NPC1 protein model (Fig. 1). Homozygous/heterozygous compound carriers of partial/complete loss-of-function coding mutations in *NPC1* result in NPC1 disease^16^. We identified four homozygous carriers of three rare *NPC1* missense mutations (p.Gly149Arg, p.Val780Met, p.Ala1108Thr) out of 122,678 participants (0.0033%). No homozygous carrier of frameshift, stop gained, essential splice site and start loss mutations was observed in gnomAD. The four homozygous carriers, three males and one female, all belong to the South Asian ethnic group (N = 15,308; homozygous carrier frequency: 0.026%). The other ethnic groups (Ashkenazi Jewish, African, East Asian, European, and Latino) had no homozygous carriers. When the sample was restricted to control individuals, only one homozygous carrier remained in the sample. However, no homozygous carrier was considered as a neurological case in gnomAD.

**Table 2:** Ethnic differences, sex distribution and global prevalence of pathogenic *NPC1* mutations.

	Global (N = 122,678)	African (N = 8128)	Ashkenazi Jewish (N = 5040)	East Asian (N = 9197)	European (N = 67,709)	Latino (N = 17,296)	South Asian (N = 15,308)
^a^Total alleles, N	245,356	16,256	10,080	18,394	135,418	34,592	30,616
^**b**^**Mutated alleles, N**	1574	265	28	107	548	251	375
Frequency, (%)	(0.64%)	(1.63%)	(0.28%)	(0.58%)	(0.40%)	(0.73%)	(1.22%)
**Heterozygous carriers, N**	1566	265	28	107	548	251	367
Frequency, (%)	(1.28%)	(3.26%)	(0.56%)	(1.16%)	(0.81%)	(1.45%)	(2.40%)
**Homozygous carries, N**	4	0	0	0	0	0	4
Frequency, (%)	(0.0033%)	(0%)	(0%)	(0%)	(0%)	(0%)	(0.026%)
**Sex, M/F**	66,355/56,323	3093/5035	2590/2450	4533/4664	37,442/30,267	7161/10,135	11,536/3772
Heterozygous carriers, M/F	813/753	96/169	8/20	48/49	296/252	106/145	259/108
Homozygous carriers, M/F	3/1	0/0	0/0	0/0	0/0	0/0	3/1
**Ethnic-specific mutations**							
Mutated alleles, N	611	44	7	46	275	107	132
Heterozygous carriers, N	609	44	7	46	275	107	130
Homozygous carriers, N	1	0	0	0	0	0	1

*M *male, *F *female.

^a^The total number of alleles in each group (determined by multiplying the number of individuals by two).

^b^Pathogenic alleles with a minor allele frequency lower than 1% in all ethnic groups.

Heterozygous carriers of partial/complete loss-of-function coding mutations in the *NPC1* gene confer an increased risk of obesity in East Asian populations^14^. We identified 1566 heterozygous carriers of rare *NPC1* mutations out of 122,678 participants (1.28%). Of the 1566 heterozygous carriers, 28 belong to the Ashkenazi Jewish ethnic group (N = 5040; heterozygous carrier frequency: 0.56%), 548 to the European ethnic group (N = 67,709; heterozygous carrier frequency: 0.81%), 107 to the East Asian ethnic group (N = 9197; heterozygous carrier frequency: 1.16%), 251 to the Latino ethnic group (N = 17,296; heterozygous carrier frequency: 1.45%), 367 to the South Asian ethnic group (N = 15,308; heterozygous carrier frequency: 2.40%), and 265 to the African ethnic group (N = 8128; heterozygous carrier frequency: 3.26%). A 5.8-fold difference was observed between the lowest and highest prevalence of heterozygous carriers.

**Figure 2:**
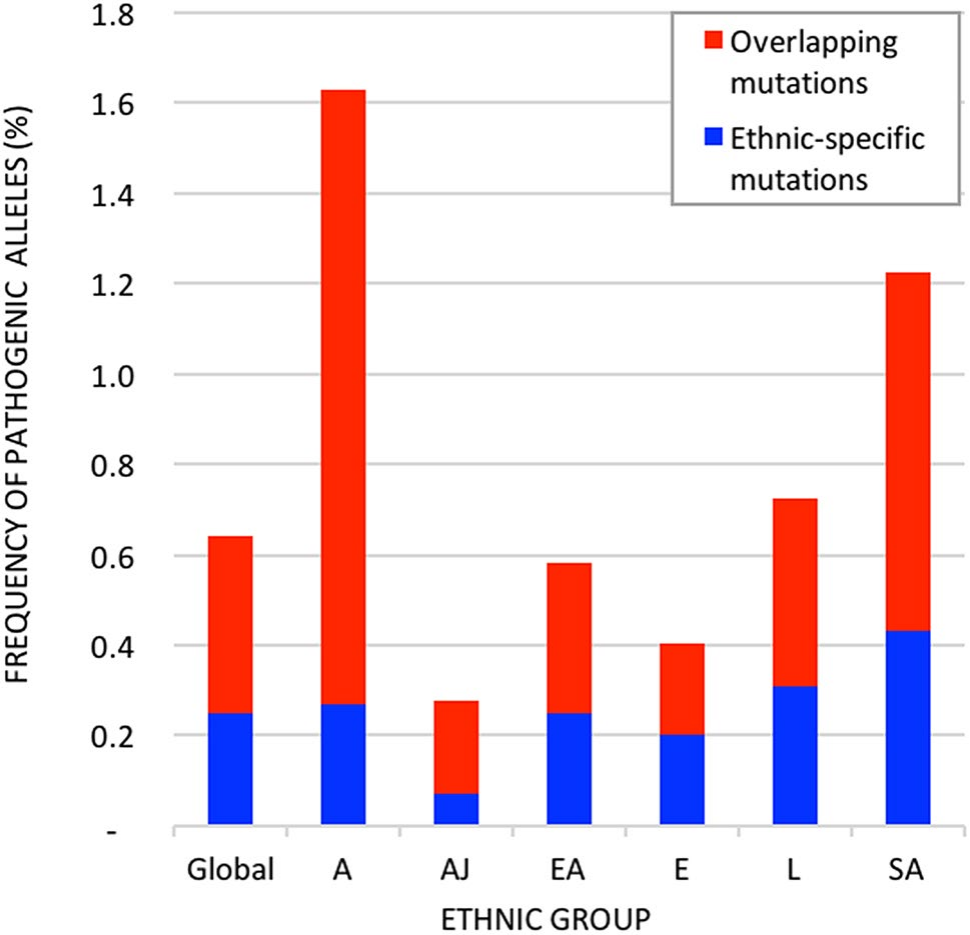
The cumulative frequency of pathogenic mutated alleles in *NPC1* globally and by ethnic groups. Ethnic-specific mutations represent mutations that are prevalent in only one ethnic group from gnomAD. Overlapping mutations are prevalent in more than one ethnic group. The global prevalence is described, as well as the prevalence for each of the six populations: Europeans (E), Latino (L), South Asian (SA), East Asian (EA), African/African American (A), and Ashkenazi Jewish (AJ).

The cumulative frequency of pathogenic mutated alleles in *NPC1* was significantly different between the six ethnic groups from gnomAD (P-value = 0), with 5.8-fold difference between the lowest and highest mutation frequency: Ashkenazi Jewish (0.28%), European (0.40%), East Asian (0.58%), Latino (0.73%), South Asian (1.22%), and African (1.63%). Of the 414 mutations, 340 mutations (82%) are unique to only one ethnic group: five belonging to the Ashkenazi Jewish population, 32 in the African population, 36 in the East Asian population, 43 in the Latino population, 57 in the South Asian population, and 167 mutations unique to the European population (Supplementary Table 1). These ethnic-specific mutations represent 609 heterozygous carriers and one homozygous carrier (p.Ala1108Thr). The distribution of ethnic-specific mutations in heterozygous and homozygous carriers of *NPC1* pathogenic mutations is significantly different between the ethnic groups (P-value = 0). The cumulative frequency of pathogenic alleles in *NPC1* is represented in Fig. 2.

### Sex differences in the prevalence of *NPC1* pathogenic mutations

Table 2 summarizes male and female distribution of *NPC1* pathogenic mutations in heterozygous and homozygous carriers. A significant difference in the prevalence of pathogenic mutations was not observed between male and female individuals globally and within the six ethnic groups. Of the 414 mutations, 149 mutations were male-specific, 143 were female-specific, and 122 mutations were present in both male and female participants. More details on these mutations can be found in Supplementary Tables 1 and 2.

## Discussion

Unexpectedly, our findings do not provide evidence for negative selection of LOF, non-synonymous, and synonymous mutations in the *NPC1* gene. Further, the pLI value of 0 may be indicative of positive natural selection for LOF mutations, although this conclusion should be interpreted with caution because the prevalence of observed LOF mutations was three times lower than the expected value. The length of the gene can influence the reliability of the pLI value where short genes are less likely to be accurately detected^32^, and considering that *NPC1* is a longer gene, the 0 value for pLI is likely to be accurate. It may be counterintuitive for LOF mutations in *NPC1* to be tolerated, considering that homozygous or heterozygous compound LOF mutations in *NPC1* cause a life-threatening recessive lipid storage disorder^16^. Further, the reproductive fitness of patients with NPC1 disease is substantially compromised due to a shorter life expectancy (10–25 years old), and neurological impairments^16^. The pLI score is more adapted to identify haploinsufficient genes that cause life-threatening dominant diseases. In the case of the *NPC1* gene, although a heterozygous mutation does not usually lead development of NPC1 disease, cases have been reported whereby a heterozygous mutation results in partial manifestation of “NPC1 heterozygous disease”^33,34^. Carriers of pathogenic *NPC1* mutations have been described to have a negative natural selection considering obesity has been linked to infertility^35^. However, human evolution has occurred during historic periods of famine^35^ when heterozygous mutations may actually positively impact fitness by providing an evolutionary advantage due to an increased ability to store fat, aligning with the ‘thrifty genotype hypothesis’^26,28^. Therefore, the *NPC1* gene may have been subject to balanced selection, where heterozygotes observed a higher reproductive fitness and homozygotes had a lower fitness, resulting in no negative signature of natural selection^26,29,30^. With respect to the 27 NPC1 protein domains, the highest percentage of mutant amino acids and individuals harboring these mutations per domain were consistent with the last three cytoplasmic domains for targeting to late endosomes, the second large luminal domain that binds NPC2, and a domain adjacent to the SSD. Therefore, these NPC1 protein domains, which are of central importance for structure and function are enriched with pathogenic mutations, may prove to be deleterious in the homozygous state and possibly advantageous in the heterozygous state assuming balancing selection.

Our study indicates that the frequency of *NPC1* pathogenic mutations differs significantly among ethnic groups, with over 80% of the mutations found in one ethnic group. In fact, the divergent pattern observed is consistent with a balanced selection model, where mutations with low and relatively equal frequencies among ethnic groups may have been negatively selected against^27^. Conversely, mutations with highly varied frequencies between ethnic groups may be explained by local patterns of positive selection^27^. Liu et al. recently described that East Asian heterozygous *NPC1* LOF mutation carriers had a significantly higher BMI and risk of severe obesity than non-carriers^14^. The prevalence of *NPC1* heterozygous carriers in our study is consistent with previously reported prevalence of obesity across different ethnic groups. Most notably, African, Latino, and South Asian populations have been reported to have a higher risk of obesity/abdominal obesity than Caucasians and East Asians, which aligns with the present findings where the highest prevalence of deleterious *NPC1* mutations was observed in the Latino, South Asian, and African groups^36,37^. *NPC1* heterozygous carriers of pathogenic mutations represent 0.56–3.26% of the gnomAD population depending on the ethnic group. Considering that these individuals have potentially a high risk to develop obesity^14^, *NPC1* may account for a non-negligible fraction of obesity cases in the general population. In fact, four heterozygous *NPC1* variants (p.Thr743Ile, p.Arg794Trp, p.Val1044Met, p.Thr1176Met) were found by Liu et al. in seven obese young patients with BMIs ranging from 35.4 to 45.4, and were also captured in our sample^14^. In addition, Lui et al. describe a heterozygous LOF *NPC1* gene mutation (p.Val780fs*) in an obese patient with a BMI of 45.6^14^, suggesting that LOF *NPC1* mutations may indeed predispose individuals to a higher BMI. Although Lie et al. report sex-specific associations between pathogenic NPC1 mutations and obesity, we found no significant difference between the prevalence of mutations in males and females. It is important to note that the study by Lui et al. was limited to a modest East Asian sample and further genotype–phenotype studies are required confirm associations with obesity in diverse ethnic groups.

We identified four homozygous carriers of three rare *NPC1* missense mutations out of 122,678 participants (0.0033%), all belonging to the South Asian ethnic group. It is possible that these individuals are in fact affected by NPC1 disease, known to be caused by homozygous or heterozygous compound *NPC1* pathogenic mutations. The prevalence of homozygous carriers in the current population (0.0033%) is consistent with the estimated prevalence of Niemann–Pick disease type C on ORPHANET (0.001–0.009%), keeping in mind that *NPC1* mutations account for 95% of disease cases^16^. Further, endogamy has been postulated to underlie a high recessive disease burden in South Asian populations^38^. The ancestral variants are non-polar and shielded from the aqueous environment due to being buried within the hydrophobic core of LDA (p.Gly149Arg) and associated with alpha helices TD7 (p.Val780Met) and TD9 (p.Ala1108Thr) within the hydrophobic core of a phospholipid bilayer. Moreover, compared to the ancestral variants, the respective derived variants possess side chains with a much larger spatial area. Therefore, since the combined homozygous mutations have different physiochemical properties, the protein secondary and tertiary structures will be adversely effected, which in turn will negatively influence thermodynamic stability resulting in a biological loss-of-function. Our findings suggest that this increased risk may also extend to NPC1 disease. To our knowledge, NPC1 disease has yet to be studied in South Asian populations. This may represent a priority for future investigations to increase our understanding of the populations at greatest risk of developing NPC1 disease. Considering that the gnomAD database attempts to exclude cases of rare pediatric life-threatening disease, our finding of four potential NPC1 cases may have several plausible explanations. These individuals may have experienced a late onset or non-fully penetrant disease manifestations which did not meet the criteria for a pediatric life-threatening disease or these cases may have simply not been captured by gnomAD. Notably, variant p.Gly149Arg has been reported as benign/likely benign for NPC1 disease in ClinVar, although clinical features of patients with the variant were not described. As for carriers of NPC1 disease, it is possible that this variant is only partially deleterious and thereby placing patients at increased risk of less penetrant disease manifestations such as obesity. These findings should be interpreted cautiously, as a conclusion cannot be made without clinical and in vitro functional characterization data confirming that these mutations (p.Gly149Arg, p.Val780Met, p.Ala1108Thr) are indeed disease causing.

The current investigation has several strengths. This is the first study to investigate the prevalence of *NPC1* mutations in diverse ethnic groups, providing a global understanding of the implications of *NPC1* in obesity risk and NPC1 disease. This is also the first investigation of a potential signature of natural selection for the *NPC1* gene. Further, conclusions are drawn from a large sample size of 122,678 participants from gnomAD, reducing bias associated with random sampling error and small sample size. This study also presents several limitations. The sample population on gnomAD is a mixture of the general population along with disease cases (e.g., type 2 diabetes, cancer, neurological conditions), therefore limiting the applicability of the current findings to the general population. The data on gnomAD is aggregated from multiple sources and the sampling methods of each study is unavailable, introducing potential bias in the population-specific results. In addition, gnomAD does not represent all ethnic groups, such as Arabs, Pacific Islanders, and Native Australians. Phenotypic data is also not accessible making it difficult to ascertain the presence and severity of disease associated with specific mutations. The pathogenicity of missense mutations was investigated using in silico prediction tools and the ClinVar database. We used SIFT and Polyphen2 as these were available in gnomAD and enabled large-scale, transparent, and reproducible findings, although we acknowledge that other in silico tools have been described with better prediction values for cholesterol transport proteins^39^. We are also aware that in vitro experiments will have enhanced the functional characterization of mutations. We also excluded *NPC1* coding synonymous mutations based on their low likelihood of pathogenicity, but we acknowledge that some of these mutations may have deleterious consequences on NPC1 expression/function^40^. Finally, the functionalities of the gnomAD database do not enable the identification of heterozygous compound mutations, and individuals affected by severe pediatric diseases have been removed, which may have resulted in an underestimation of the prevalence of suspected NPC1 disease cases.

In summary, we did not identify a negative selection for pathogenic mutations in the *NPC1* gene. This result is consistent with a balanced selection model, where *NPC1* heterozygotes display a higher reproductive fitness and homozygotes have a lower fitness. Our study shows that the frequency of *NPC1* pathogenic mutations differs significantly among ethnic groups. Depending on the ethnic group, between 0.56% and 3.26% of the gnomAD population is heterozygous for *NPC1* pathogenic mutations that may result in a high-risk of obesity. In addition, homozygous carriers of *NPC1* pathogenic mutations that can result in NPC1 disease were only found in the South Asian population (0.026%). Our study contributes to a better understanding of the global impact of *NPC1* mutations in human diseases.

## Subjects and methods

### Participants

The dataset on gnomAD v2.1.1 includes 141,456 unrelated individuals (125,748 exomes and 15,708 genomes) sequenced to perform population-genetic and disease-specific studies. Of these individuals, sub-samples were defined by gnomAD: 60,146 samples as controls (not a case in a case/control study of common disease), 134,187 samples as non-cancer (not a case in a cancer study), 114,704 samples as non-neuro (not a case in a case/control study of neurological disease). Individuals affected by severe pediatric diseases were removed. We chose to only include the exome data of the complete gnomad v2.1.1 dataset in our analysis to keep the sample size stable for estimating the prevalence of mutations. Exome sequenced participants in gnomAD have been grouped in seven ethnic groups using a principal component analysis to distinguish the major axes of geographic ancestry: Europeans (N = 67,709), Latino (N = 17,296), South Asians (N = 15,308), East Asians (N = 9197), Africans/African Americans (N = 8128), Ashkenazi Jewish (N = 5040) and other (N = 3070). As the category ‘other’ is comprised of several ethnicities and does not provide details on the geographic origin of individuals, we did not include them in our analyses. Additional details regarding the sample populations have been described previously^32,41^. The data was collected from gnomAD on April 17th, 2020. Research was conducted in accordance with relevant guidelines and regulations of the Declaration of Helsinki and was approved by all the local institutional review boards of studies participating to ExAC and gnomAD (e.g. Broad Institute), as previously described^32,41^. Written informed consent was obtained from each subject prior to participation.

### Sequencing data quality control, release, and reporting in ExAC and gnomAD

Full details of data processing, variant calling, filtering process and variant annotation in ExAC have been previously described^32^. GnomAD was quality controlled and analyzed using the Hail open source framework (https://github.com/hail-is/hail)^41^. This data set can be accessed via the gnomAD Browser (https://gnomad.broadinstitute.org/).

### Functional characterization of *NPC1* coding mutations

Isoform I of the NPC1 protein is the canonical sequence and therefore all reported protein consequences in the manuscript correspond to isoform I. We only included *NPC1* mutations that displayed a minor allele frequency lower than 1% in all ethnic groups. Nonsense, splice acceptor, splice donor, and frameshift mutations were considered as LOF. Whether non-synonymous mutations significantly affected protein function was investigated by the SIFT and PolyPhen2 software. We previously showed that Polyphen2 predictions are comparable to the conclusions resulting from in vitro tests for the *PCSK1* gene^42^. We also included *NPC1* coding mutations reported as pathogenic/likely pathogenic in the ClinVar database^43^. Synonymous coding mutations were not considered pathogenic. The current parameters in gnomAD that were used to assess pathogenicity were not available for in frame insertions/deletions so these mutations were excluded from further study. Mutations flagged by gnomAD as dubious variant annotation or quality were not considered. Pathogenic mutations present in the ‘other’ ethnicity group and mutations with protein consequences not corresponding to isoform 1 were also excluded.

### Evolutionary genetic analyses

Lek et al. has established the probability of each gene being LOF (non-sense and essential splice site) intolerant (pLI) using the expectation–maximization (EM) algorithm in ExAC^32^. A pLI ≤ 0.1 indicates that the gene is LOF tolerant, meaning there is no substantial negative selection against loss-of-function mutations. A pLI ≥ 0.9 indicates that the gene is LOF intolerant. Lek et al. created Z scores to establish the significance of the deviation of observed synonymous and non-synonymous variant counts per gene from expectation. Significantly elevated synonymous and non-synonymous counts corresponded to Z scores < − 3.71 and < − -3.09, respectively. Significantly depleted synonymous and non-synonymous counts corresponded to Z scores > 3.71 and > 3.09, respectively. These Z-score values are equivalent to a P-value of 10^–3^ and represent the significance threshold when splitting transcripts into constrained and unconstrained classes^32^. According to the gnomAD browser information, an observed/expected mutation ratio 90% confidence interval upper bound value < 0.35 is indicative of strong mutation intolerance.

### Statistical analyses

The comparison of pathogenic mutation frequencies in *NPC1* and sex distributions among ethnic groups in gnomAD was conducted using Chi-square tests. All reported *P*-values are two-sided. *P*-values of less than 0.05 were considered significant.

### Compliance with ethical standards

Written informed consent was obtained from each participant before participation, in accordance with the Declaration of Helsinki.

## Supplementary information

Supplementary Table 1.

Supplementary Table 2.

## Data Availability

The data that supports this investigation are publicly and openly available in the Genome Aggregation Database V 2.1.1 at https://gnomad.broadinstitute.org/.
