# Supplementary material for: Signatures of natural selection and ethnic-specific prevalence of NPC1 pathogenic mutations contributing to obesity and Niemann–Pick disease type C1
Source: Sci Rep. 2020 Nov 2;10:18787. doi: 10.1038/s41598-020-75919-4 (PMC7608643; doi:10.1038/s41598-020-75919-4)
Supplement: Supplementary file 1 — Supplementary Table 1. [file 41598_2020_75919_MOESM1_ESM.docx]

**Signatures of natural selection and ethnic-specific prevalence of *NPC1* pathogenic mutations contributing to obesity and Niemann-Pick disease type C1**

**Andreea Chiorean^1^, William S. Garver^2^, David Meyre^1,3*^**

^1^Department of Health Research Methods, Evidence, and Impact, McMaster University, Hamilton, ON, Canada; ^2^Department of Chemistry and Chemical Biology, University of New Mexico, Albuquerque, NM, United States of America; ^3^Department of Pathology and Molecular Medicine, McMaster University, Hamilton, ON, Canada.

ORCID iD

Andreea Chiorean 0000-0003-4088-4069

William S. Garver 0000-0002-9635-8474

David Meyre 0000-0003-4850-7444

*Correspondence: Dr D Meyre, Department of Health Research Methods, Evidence, and Impact, McMaster University, 1280 Main Street West, Michael G. DeGroote Centre for Learning and Discovery Room 3205, Hamilton, ON L8S 4K1, Canada.

E-mail: meyred@mcmaster.ca Fax: 905-528-2814 Phone: 905-525-9140 ext. 26802

Supplementary Table 2. Protein domain location of 355 missense *NPC1* mutations

| **NPC1 Protein Domain (AA)** | **N Mutations** | **Mutations Per Domain**  **(% AA)** | **N Individuals**  **(%Male)** | **Individuals Per**  **Domain**  **(% AA)** | **N Male-Specific Mutations** | **N Female-Specific Mutations** |
| --- | --- | --- | --- | --- | --- | --- |
| **LDA: AA 1-269 (269)** | 46 | 17.10% | 304 (172/134) | 113.01% | 15 | 18 |
| **TD1: AA 270-290 (21)** | 2 | 9.52% | 9 (4/5) | 42.86% | 1 | 0 |
| **CDB: AA 291-350 (60)** | 11 | 18.33% | 24 (15/9) | 40.00% | 6 | 3 |
| **TD2: AA 351-371 (21)** | 1 | 4.76% | 10 (4/6) | 47.62% | 0 | 0 |
| **LDC: AA 372-619 (248)** | 73 | 29.44% | 383 (179/204) | 154.44% | 28 | 24 |
| **TD3: AA 620-640 (21)** | 5 | 23.81% | 8 (6/2) | 38.10% | 3 | 1 |
| **CDD: AA 641-655 (15)** | 6 | 40.00% | 10 (4/6) | 66.67% | 2 | 2 |
| **TD4: AA 656-675 (20)** | 11 | 55.00% | 19 (8/11) | 95.00% | 2 | 6 |
| **LDE: AA 676-678 (3)** | 1 | 33.33% | 1 (0/1) | 33.33% | 0 | 1 |
| **TD5: AA 679-697 (19)** | 5 | 26.32% | 7 (4/3) | 36.84% | 3 | 1 |
| **CDF: AA 698-735 (38)** | 12 | 31.58% | 34 (19/15) | 89.47% | 8 | 2 |
| **TD6: AA 736-755 (20)** | 6 | 30.00% | 9 (6/3) | 45.00% | 3 | 2 |
| **LDG: AA 756-760 (5)** | 1 | 20.00% | 2 (0/2) | 40.00% | 0 | 1 |
| **TD7: AA 761-780 (20)** | 11 | 55.00% | 31 (21/10) | 155.00% | 4 | 5 |
| **CDH: AA 781-833 (53)** | 18 | 33.96% | 51 (26/26) | 96.23% | 2 | 12 |
| **TD8: AA 834-853 (20)** | 6 | 30.00% | 25 (13/12) | 125.00% | 1 | 2 |
| **LDI: AA 854-1097 (244)** | 69 | 28.28% | 288 (159/129) | 118.03% | 24 | 18 |
| **TD9: AA 1098-1118 (21)** | 7 | 33.33% | 12 (6/6) | 57.14% | 2 | 2 |
| **CDJ: AA 1119-1125 (7)** | 3 | 42.86% | 12 (7/5) | 171.43% | 1 | 1 |
| **TD10: AA 1126-1145 (20)** | 4 | 20.00% | 4 (2/2) | 20.00% | 2 | 2 |
| **LDK: AA 1146-1148 (3)** | 1 | 33.33% | 1 (1/0) | 33.33% | 1 | 0 |
| **TD11: AA 1149-1167 (19)** | 7 | 36.84% | 11 (6/5) | 57.89% | 3 | 3 |
| **CDL: AA 1168-1196 (29)** | 16 | 55.17% | 128 (68/60) | 441.38% | 2 | 5 |
| **TD12: AA 1197-1216 (20)** | 8 | 40.00% | 17 (8/9) | 85.00% | 1 | 2 |
| **LDM: AA 1217-1227 (11)** | 4 | 36.36% | 5 (3/2) | 45.45% | 2 | 1 |
| **TD13: AA 1228-1248 (21)** | 4 | 19.05% | 11 (6/5) | 52.38% | 1 | 1 |
| **CDN: AA 1249-1278 (30)** | 17 | 56.67% | 53 (18/35) | 176.67% | 4 | 7 |
| **TOTAL** | **355** | **-** | **1469 (764/705)** | **-** | **121** | **122** |

AA = amino acids

LD = luminal domains

CD = cytoplasmic domains

TD = transmembrane domains
